# The Burden of Impaired Serum Albumin Antioxidant Properties and Glyco-Oxidation in Coronary Heart Disease Patients with and without Type 2 Diabetes Mellitus

**DOI:** 10.3390/antiox11081501

**Published:** 2022-07-30

**Authors:** Francesco Piarulli, Cristina Banfi, Maura Brioschi, Alessandra Altomare, Eugenio Ragazzi, Chiara Cosma, Giovanni Sartore, Annunziata Lapolla

**Affiliations:** 1 Department of Medicine, University of Padova, 35128 Padova, Italy; francesco.piarulli@unipd.it (F.P.); chiara.cosma@aopd.veneto.it (C.C.); g.sartore@unipd.it (G.S.); 2 Centro Cardiologico Monzino IRCCS, 20138 Milan, Italy; maura.brioschi@ccfm.it; 3 Department of Pharmaceutical Sciences, University of Milan, 20133 Milan, Italy; alessandra.altomare@unimi.it; 4 Department of Pharmaceutical and Pharmacological Sciences (DSF), University of Padova School of Medicine and Surgery, 35131 Padova, Italy; eugenio.ragazzi@unipd.it

**Keywords:** diabetes, S-thiolation, oxidative stress, albumin, coronary heart disease

## Abstract

Human serum albumin (HSA) has an important antioxidant activity due to the presence of the reduced cysteine at position 34, which represents the most abundant free thiol in the plasma. In oxidative-based diseases, HSA undergoes S-thiolation (THIO-HSA) with changes in the antioxidant function of albumin that could contribute to the progression of the disease. The aim of this study was to verify, for the first time, the different burdens of THIO-HSA, glycated HSA (GLY-HSA), and advanced glycation end products (AGE) accumulation both in type 2 diabetes mellitus (T2DM) patients and in non-diabetic patients, with or without coronary heart disease (CHD). In this study, we assessed the presence of modified forms of HSA, THIO-HSA, and GLY-HSA by means of mass spectrometry in 33 patients with both T2DM and CHD, in 31 patients with T2DM and without CHD, in 30 patients without diabetes with a history of CHD, and 27 subjects without diabetes and CHD. All the patients’ anthropometric and clinical data were recorded including age, sex, duration of diabetes, body mass index (BMI), blood pressure, and history of CHD defined with anamnestic data. Metabolic parameters, such as fasting plasma glucose (FPG), glycated hemoglobin (HbA1c), lipids, pentosidine, AGE, receptor for advanced glycation end-products (RAGE) and its soluble form (sRAGE), were measured. AGE and pentosidine are significantly higher in T2DM patients with and without CHD with respect to non-diabetic patients with CHD and control subjects. RAGE levels are significantly higher in T2DM patients with respect to non-diabetic patients, and among T2DM patients, the group with CHD showed significantly higher RAGE levels than those without CHD (217 ± 171 pg/mL and 140 ± 61 pg/mL, respectively). Albumin isoforms discriminate between non-diabetic patients with CHD and T2DM patients with and without CHD and control subjects, with GLY-HSA levels higher in T2DM with and without CHD, and THIO-HSA higher in CHD patients without T2DM. Finally, we demonstrated that the oxidized forms of HSA can increase the expression of the inflammatory cytokine Tumor Necrosis Factor-alpha (TNFα) in monocytic cells. In patients with CHD, GLY-HSA and THIO-HSA have a different prevalent distribution, the first one prevailing in patients with T2DM and the second one in patients without T2DM. These findings suggest that albumin quality and homeostasis balance between glyco-oxidation and thiolation might have an impact on the antioxidant defense system in cardiovascular diseases.

## 1. Introduction

Type 2 diabetes mellitus (T2DM) is associated with a two- to four-fold increase in the risk of coronary, cerebral, and peripheral artery disease [1]. A series of biochemical alterations due to hyperglycemia, including protein kinase C activation, an increase in polyol and hexosamine pathway flux, and non-enzymatic protein glycation are also involved in atherosclerotic disease development [2,3]. All of these molecular mechanisms reflect a single hyperglycemia-induced process of superoxide overproduction by the mitochondrial electron transport chain [4]. Hyperglycemia and increased oxidative stress act through common pathways leading to tissue damage [4]. As for protein glycation, this process is the first of a series of steps, leading to the production of advanced glycation end products (AGE). Pentosidine is one of the most investigated AGE, which results from the non-enzymatic reactions between proteins and pentoses or hexoses. It is a very sensitive marker of chronic diseases, and it is directly associated with early and advanced atherosclerosis in patients with diabetes mellitus [5]. AGE accumulation is able to determine endothelial damage through a series of mechanisms, including intracellular protein modifications, formation of cross-links in the extracellular matrix, and interaction with the AGE receptor, RAGE [6]. RAGE is a multiligand member of the Ig superfamily of cell surface molecules that binds AGE and induces cellular signaling, including activation of the nuclear factor κB (NF-κB), an increase in cytokine and adhesion molecule expression, and induction of oxidative stress, with an increase in cytosolic reactive oxygen species [7,8]. AGE and their receptors may be useful biomarkers for the presence and severity of coronary heart disease (CHD) in patients with and without diabetes mellitus; the AGE/RAGE axis has been implicated in contributing to cardiovascular mortality independently of diabetes [9]. The soluble RAGE form (sRAGE) reflects the total concentration of RAGE present in cells and plasma [3]. In patients with CHD, some clinical studies have demonstrated that lower levels of plasma sRAGE are inversely associated with the severity of CHD [10]. However, other reports have shown that serum sRAGE levels are significantly higher in patients with T2DM than in non-diabetic subjects, and are positively associated with the presence of CHD [11].

Human serum albumin (HSA) is the most abundant circulatory protein, and has an important antioxidant activity by trapping free radicals [12]. This protein exerts this activity through the presence of the reduced Cys 34 [13]; the Cys 34 sulphydryl group present in the albumin is the largest fraction of all free thiols in plasma, and this confers to HSA a major role in the antioxidant capacity of the plasma [14]. In a previous paper, Brioschi et al. evidenced that S-thiolation of albumin (THIO-HSA) is increased in the plasma of patients with heart failure (HF) and induces changes in the antioxidant function of albumin that could contribute to the progression of HF [15]. An enhanced THIO-HSA was observed also in the plasma of hemodialysis patients [16], suggesting its possible role in inducing changes in biological responses in the inter-cellular and inter-organ traffic, leading to the development of chronic complications. Moreover, low HSA levels are independently and inversely associated with the occurrence of cardiovascular diseases (CVD) including HF, CHD, atrial fibrillation, stroke, and venous thromboembolism, representing a potential risk factor for various CVD [12]. On the other side, as demonstrated by studies made with mass spectrometry (MS) [17,18], HSA can undergo glycation (GLY-HSA), both in diabetes mellitus and in non-diabetic patients affected by HF [15,19]. GLY-HSA is now considered a clinical biomarker of glycemic control, even if its use is restricted to those clinical conditions in which the levels of glycated hemoglobin (HbA1c) do not reflect glycemic status (i.e., pregnancy, chronic renal failure, or transfusion), or to the evaluation of short-term glycemic fluctuations in patients with poor metabolic control. Indeed, GLY-HSA reflects mean glycemia over approximately 2–3 weeks and, compared with HbA1c is not influenced by red blood cell lifespan [20]. However, as stated by the International and National Recommendations, it cannot be used yet for the diagnosis of diabetes [21,22].

The aim of this study was to verify, for the first time, the different burdens of THIO-HSA, GLY-HSA, and AGE accumulation both in T2DM patients and in non-diabetic patients, with or without CHD.

## 2. Materials and Methods

### 2.1. Patients Recruitment and Clinical Parameters

Thirty-three patients with both T2DM and CHD (group A), 31 patients with T2DM and without CHD (group B), 30 patients without diabetes with a diagnosis of CHD (group C), and 27 subjects without diabetes and CHD (group D) were examined. All patients affected by diabetes (groups A and B) followed an isocaloric Mediterranean-style dietary pattern (characterized by a lower intake of proteins from animal food sources, saturated fat and cholesterol, added sugars, a higher intake of fiber, and a lower glycemic index and glycemic load), and had comparable hypoglycemic therapy. In particular, no difference occurred with the use of metformin, which was administered in a small number of patients (5 patients in group A, and 4 patients in group B), and in low doses (500–750 mg/day). On the day of the study, all the patients’ anthropometric and clinical data were recorded including age, sex, body mass index (BMI), blood pressure, and diagnosis of CHD defined with anamnestic data from electronic medical records. Standard criteria for disease diagnosis were followed for the inclusion of patients into the groups investigated. CHD was assessed from the hospital records; 90% of the patients were taking antihypertensive drugs. The study was conducted in accordance with the Declaration of Helsinki and its later amendments, and was approved by the Ethics Committee for Clinical Trials in the Province of Padova, reference study No. 97610. Informed written consent was obtained from all subjects participating in the study.

Blood samples were collected in tubes containing citrate as the anticoagulant (0.129 mmol/L). Plasma was immediately prepared by means of centrifugation at 1500× *g* for 15 min at 4 °C, divided into aliquots, and frozen at −80 °C until assayed.

Metabolic parameters, such as fasting plasma glucose (FPG), HbA1c, total cholesterol, HDL and LDL cholesterol, and triglycerides were evaluated, and glycation and oxidation parameters such as GLY-HSA, THIO-HSA, pentosidine, AGE, sRAGE were measured. FPG was measured using the glucose oxidase method [23]. HbA1c was measured using an International Federation of Clinical Chemistry (IFCC)-aligned standardized HPLC method [24]. Plasma lipids were measured as proposed by Dastych et al. [25].

### 2.2. Immunoassays

Plasma levels of sRAGE, AGE [26], Pentosidine, and RAGE were measured by enzyme-linked immunosorbent assays (ELISA), according to the manufacturer’s instruction.

Soluble RAGE was measured with an ELISA kit produced by BioVendor Research and Diagnostic on plasma samples. A calibration curve (50–3200 pg/mL) was used to calculate sRAGE plasma concentrations. The AGE ELISA kit was purchased from Cusabio and was used to measure AGE levels using the provided calibration curve (0.78 µg/mL–50 µg/mL) for calculation of the concentration. Human pentosidine plasma levels, measured with the ELISA kit produced by Cusabio, were calculated using the provided standard curve (31.25 pmol/mL–2000 pmol/mL). The Human RAGE ELISA kit produced by RayBiotech was used to measure plasma RAGE levels, and concentration was calculated from the calibration curve (3 pg/mL–1500 pg/mL). Absorbance was measured with the Tecan infinite M200 spectrophotometer.

### 2.3. Albumin Analysis by Mass Spectrometry

Liquid chromatography-mass spectrometry (LC-MS) analysis of intact proteins in plasma was used to measure the relative abundance (%) of GLY-HSA and THIO-HSA, as previously detailed [27]. Briefly, plasma proteins were diluted 1:40 in a denaturing mobile phase (30% acetonitrile:0.1% formic acid, FA) before LC separation on a reversed-phase column Jupiter C4 (150 × 2 mm, i.d. 5 µm, 300 Å, Phenomenex, Milan, Italy) by means of a 20 min gradient using 0.1 % FA in water as the mobile phase A and 0.1% FA in acetonitrile as the mobile phase B (30% B for 2 min; 30–50% B in 11 min; 50–95% B in 1 min; 95%B for 3 min; and then 3 min at 30% B). An LC system coupled with a triple-quadrupole mass analyzer (Finnigan TSQ Quantum Ultra, ThermoQuest, Milan, Italy) equipped with an electrospray source was set as reported by Altomare et al. [27]. Xcalibur software was used for MS spectra acquisition. The relative abundance of HSA isoforms, based on their corresponding peak area, was computationally calculated by using MagTran software, designed to perform deconvolution of the acquired multi-charge spectra [27].

### 2.4. Regeneration of Mercaptoalbumin (HSA-SH)

Regeneration of native albumin (mercaptoalbumin, HSA-SH) was performed by treating human serum albumin (Albutein^®^, Grifols Italia, Milan, Italy) with *N*-acetyl-cysteine (NAC) (100 µg/mL) as previously described [27]. The reaction mixtures were incubated for 1 h at 37 °C under stirring (85 rpm) in a thermomixer and then dialyzed against saline solution (NaCl, 0.9%, pH 7) to remove NAC. The amount of HSA-HS was evaluated by mass spectrometry as described above.

### 2.5. Albumin Cysteinylation

THIO-HSA was obtained following incubation of HSA (Albutein^®^, Grifols Italia, Milan, Italy) with 17 mmol/L of cysteine at 37 °C for 2 h. The solution was then dialyzed against saline solution (NaCl, 0.9%, pH 7), and immediately used for incubation with cells. The level of S-thiolation was checked by mass spectrometry as described above.

### 2.6. Cell Treatment with Albumin Isoforms

Monocytic U937 cells (American Type Tissue Culture Collection, Manassas, VA, USA) were cultured in RPMI (Euroclone, Milan, Italy) and supplemented with 10% fetal calf serum (Euroclone, Milan, Italy) at 37 °C in a humidified atmosphere containing 95% air/5% CO_2_. Before treatment, cells were incubated in a serum-free medium for 4 h and then for 2 h with regenerated mercaptoalbumin (HSA-SH), S-thiolated albumin (THIO-HSA), and glycated HSA (GLY-HSA, Sigma-Aldrich, Milan, Italy, cod A8301).

### 2.7. Real-Time Quantitative Reverse Transcriptase PCR

Total cellular RNA was extracted with the Total RNA Purification Kit (Norgen Biotek Corp.) and reverse transcribed (1 μg) as previously described [28]. The quality of RNA was checked by the Agilent 2100 Bioanalyzer system. 18S ribosomal RNA was used as a housekeeping gene to correct for RNA levels input and efficiency of the reactions. Real-time qRT-PCR was performed in triplicate with 2.5 μL of cDNA incubated in 22.5 μL IQ Supermix containing primers and SYBRGreen fluorescence dye (Bio-Rad Laboratories, Milan, Italy) using the iCycler Optical System (Bio-Rad Laboratories, Milan, Italy). The sequences of primers used as normalizers were: human 18S forward: 5′-CGG CTA CCA CAT CCA AGG AA-3′; human 18S reverse: 5′-CCT GTA TTG TTA TTT TTC GTC ACT ACC T-3′; TNF alpha Quantitect Primer Assay from Qiagen (QT00029162). Expression levels were calculated by Ct values normalized to 18S rRNA.

### 2.8. TRAP Assay

Plasma antioxidant activity was evaluated by means of the fluorimetric TRAP assay monitoring the oxidation of the substrate 2′,7′-dichlorodihydrofluorescein diacetate (DCFH2-DA) to 2,7 dichlorofluorescein (DCF), in the presence of the radical initiator, AAPH, as previously described [15]. The antioxidant activity was expressed as the lag-phase (min) induced before the substrate oxidation.

### 2.9. Statistical Analysis

Continuous variables are expressed as the mean ± standard deviation (SD). Comparison among groups was performed by one-way analysis of variance (ANOVA) followed by post hoc Tukey’s honest significance test (HSD). The correlation between parameters was evaluated by means of Pearson’s *r* correlation coefficient. Statistical significance was set at *p* < 0.05.

The sample size was chosen to guarantee a power of 80% on the determination of significant differences equal to one standard deviation with an alpha value of 0.05.

## 3. Results

**Figure 1:**
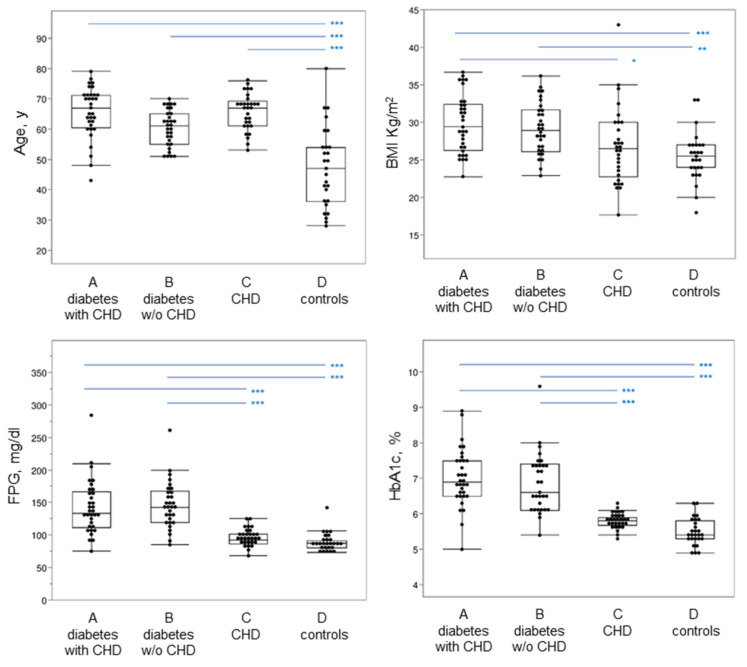
Clinical and metabolic data evaluated in the four groups of subjects. In the box plot, the ends of the box represent the 1st and 3rd quartile, respectively. Each box is divided by a line, which represents the median value. The whiskers show the values corresponding to [1st quartile−1.5 × (interquartile range)], and [3rd quartile + 1.5 × (interquartile range)]. Horizontal blue lines on the top part of the graph indicate post hoc comparisons between groups by Tukey’s HSD test, following significant ANOVA. BMI, body mass index; CHD, coronary heart disease; FPG, fasting plasma glucose; HbA1c, glycated hemoglobin. *** *p* < 0.001; ** *p* < 0.01; * *p* < 0.05.

The clinical and metabolic characteristics of the patients under study are shown in Figure 1 and Supplementary Table S1, evidencing in T2DM significantly higher values of FPG and HbA1c with respect to non-diabetic patients with CHD and control subjects. No significant differences were found concerning the LDL parameter between diabetic and non-diabetic patients with coronary heart disease.

**Figure 2:**
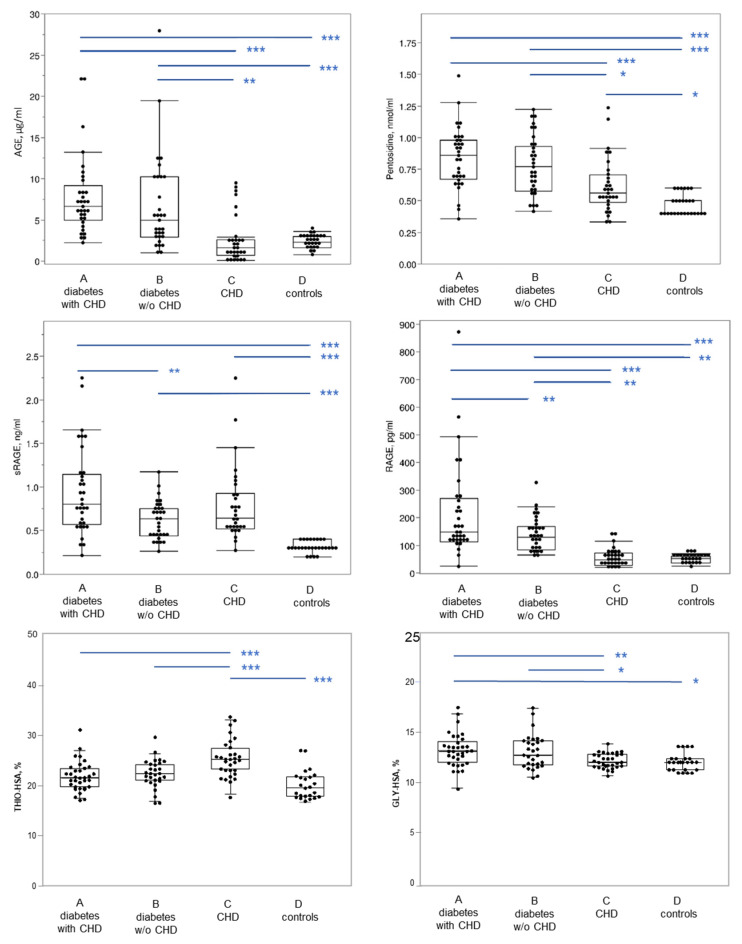
Glycation and oxidation parameters evaluated in the four groups of subjects. In the box plot, the ends of the box represent the 1st and 3rd quartile, respectively. Each box is divided by a line, which represents the median value. The whiskers show the values corresponding to [1st quartile − 1.5 × (interquartile range)], and [3rd quartile + 1.5 × (interquartile range)]. Horizontal blue lines on the top part of the graph indicate post hoc comparisons between groups by Tukey’s HSD test, following significant ANOVA. AGE, advanced glycation end products; GLY-HSA, glycated albumin; RAGE, receptor for advanced glycation end-products; sRAGE, soluble RAGE; THIO-HSA, thiolated albumin; CHD, coronary heart disease *** *p* < 0.001; ** *p* < 0.01; * *p* < 0.05.

In Table 1 and Figure 2, the advanced glycation and the oxidation parameters evaluated are reported, showing that mean values of AGE and pentosidine are significantly higher in T2DM patients with and without CHD with respect to non-diabetic patients with CHD and control subjects; no differences among the two classes of diabetic patients were found. RAGE levels are significantly higher in T2DM patients with respect to non-diabetic patients. Among T2DM patients, the group with CHD showed significantly higher RAGE levels than that without CHD. Furthermore, sRAGE levels were similar in coronaropathic patients with and without diabetes mellitus, and higher than in control subjects.

GLY-HSA levels were higher in T2DM with and without CHD with respect to non-diabetic patients with CHD and control subjects. Finally, THIO-HSA levels were higher in non-diabetic patients with CHD with respect to T2DM with and without CHD and control subjects.

**Figure 3:**
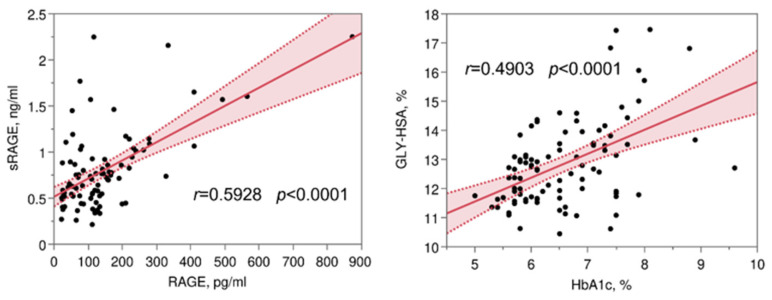
Scatter plot of variables presenting the most relevant correlations among those reported in Supplementary Table S2. Each plot shows a fitted linear regression line with a shaded 95% confidence interval. GLY-HSA, glycated albumin; RAGE, receptor for advanced glycation end-products; sRAGE, soluble RAGE; HbA1c, glycated hemoglobin.

When considering all the patients, positive significant correlations were found between AGE and FPG, HbA1c, RAGE (*r* 0.29, *p* = 0.004; *r* 0.28, *p* = 0.007; *r* 0.38, *p* = 0.0002; respectively), but not for sRAGE. A positive correlation was also found between RAGE and sRAGE (*r* 0.59, *p* < 0.0001), and between GLY-HSA and FPG and HbA1c (*r* 0.37, *p* = 0.0002; and *r* 0.49, *p* < 0.0001, respectively) (Figure 3). Furthermore, significant negative correlations were found between THIO-HSA and FPG, HbA1c, and GLY-HSA (Supplementary Table S2). Considering separately each group a positive correlation was found between AGE and THIO-HSA only in T2DM with CHD (*r* 0.38, *p* = 0.029) (Supplementary Table S2).

To verify if the age difference may have somehow influenced the results, we have stratified the healthy control group D by age, according to younger/older subjects with a cut-off of 50 years. Comparable values of all the parameters of glyco-oxidation (no significant difference) are found, confirming that age by itself is not a confounding factor in our model.

**Figure 4:**
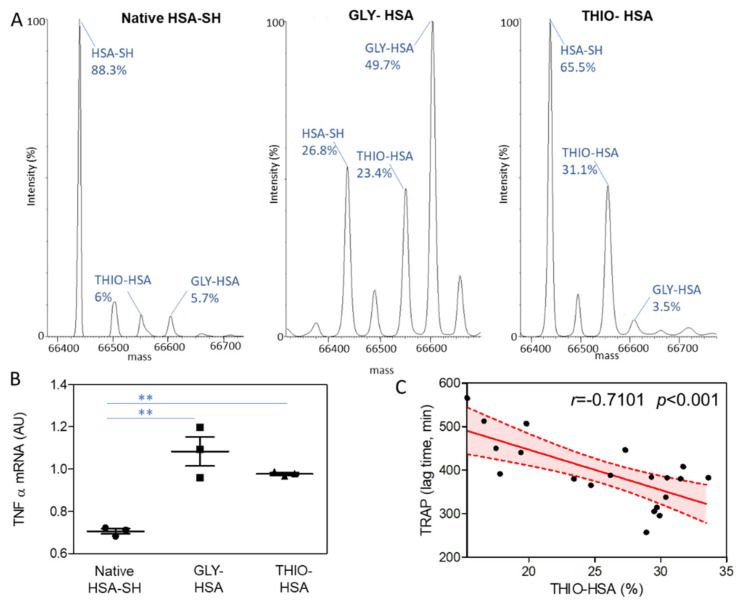
Albumin isoforms and functional effects. (**A**) Mass spectrometry analysis of regenerated HSA (native HSA-SH), highly glycated albumin (GLY-HSA), and highly thiolated albumin (THIO-HSA). (**B**) TNFα mRNA levels, normalized to the housekeeping gene 18S rRNA, in U937 cells incubated with native HSA-SH, GLY-HSA, and THIO-HSA 100 µg/mL for 2 h. ** *p* < 0.01 vs HSA-SH. (**C**) Correlation of THIO-HSA and plasma antioxidant activity measured by means of TRAP assay. Plot shows fitted linear regression line with shaded 95% confidence interval.

In order to assess the potential contribution of the different albumin isoforms in the cell inflammatory state, the monocytic cell line U937 was incubated with HSA-SH, THIO-HSA, and GLY-HSA, and the expression levels of the pro-inflammatory cytokine tumor necrosis factor-alpha (TNFα) was evaluated by real-time qRT-PCR. Mass spectrometry analysis of HSA after regeneration with NAC, and treatment with cysteine, revealed that in the regenerated albumin HSA-SH was the predominant form (88.3%), cysteine-treated HSA contained 31.1% of THIO-HSA, and GLY-HSA was characterized by a higher percentage of glycated albumin (49.7%) (Figure 4A). The incubation of U937 cells with HSA redox isoforms, THIO-HSA and GLY-HSA, significantly induced the mRNA levels of TNFα with respect to HSA-SH (Figure 4B).

In addition, we evidenced a significant negative correlation between the percentage of THIO-HSA and the total antioxidant activity in plasma, measured by means of the TRAP assay in terms of lag time before substrate oxidation (Figure 4C).

## 4. Discussion

Both glyco-oxidation parameters, AGE, and pentosidine, correlate with FPG and HbA1c and are elevated only in T2DM patients with and without CHD, and appear to induce greater plaque RAGE expression in diabetic patients with CHD, as previously described in a post-mortem study [29]. Furthermore, the positive correlation of THIO-HSA with AGE in T2DM with CHD would suggest a possible synergic effect of glyco-oxidative damage with a reduced HSA antioxidant activity on the vascular wall.

The pool of sRAGE was found unaffected by the presence of diabetes and AGE levels, while it resulted to be elevated in both T2DM and non-diabetics with CHD, suggesting that plasma levels of sRAGE may be associated with plaque vulnerability, in patients with CHD, independently from RAGE levels, according to previous studies [30,31].

In our study, we evidenced higher levels of GLY-HSA in T2DM in comparison to non-diabetics, regardless of the presence or absence of chronic cardiovascular complications, in line with its potential role as a clinical biomarker of glycemic control. An increased GLY-HSA content has been recently detected in HF patients with respect to age-matched control subjects, by Martinez Fernandez et al. with higher levels of GLY-HSA in subjects with the most severe HF [19]. The authors also demonstrated the pro-inflammatory and pro-oxidant effects of GLY-HSA on murine HL-1 cardiomyocytes. Based on these recent findings, GLY-HSA might be considered not only a potential biomarker but also a functional mediator of disease progression.

In a previous study [15], we found that THIO-HSA, derived from the binding of both cysteine and homocysteine to HSA, is increased in the plasma of patients with HF. The finding that the levels of THIO-HSA correlate with an alteration of the plasma antioxidant activity, suggests that S-thiolation induced structural and functional changes in HSA which likely contribute to the progression of the disease. Indeed, we have also provided evidence that THIO-HSA is not protective against the cytotoxic effect of oxidant hydrogen peroxide in cardiomyocytes HL-1. Thus, we hypothesized that THIO-HSA might also represent a potential causal factor in HF progression.

The results of this study suggested that THIO-HSA in non-diabetic patients with CHD, and not in T2DM with/without CHD, could represent a disease marker, similarly to HF previously described. In T2DM, the evidence that THIO-HSA levels were inversely correlated with short (FPG, GLY-HSA), and long-term (HbA1c) metabolic compensation may mask the THIO-HSA role as a marker of CHD in this population. On the other hand, the positive correlation of THIO-HSA with AGE in T2DM with CHD would suggest a possible synergic effect of glyco-oxidative damage on the vascular wall.

Finally, we demonstrated that both THIO-HSA and GLY-HSA can increase the expression of TNFα, one of the most important pro-inflammatory cytokines, in the monocytic cell line U937, thus supporting the hypothesis that post-translational modifications occurring on HSA can contribute to the progression of the disease. Although this finding is confirmatory of previous studies on the effects of GLY-HSA [32], the pro-inflammatory activity of THIO-HSA is of novelty. Indeed, besides our previous study on HF [15] and a study on hyperlipidemic subjects [33], the levels of THIO-HSA in T2DM with and without CHD have never been addressed. Regrettably, the commercial GLY-HSA contains a significant amount of THIO-HSA; thus, we cannot rule out the specific contribution of the GLY-HSA alone. On the other hand, we cannot demonstrate that the in vitro proposed mechanism is operative *in vivo*, even if we used HSA with a percentage of native HSA similar to that measured in patients and associated with a reduced total plasma antioxidant activity.

A limitation of the present investigation is the observational nature of the data that derive from subjects attending the local outpatient diabetes clinic, and therefore is representative of a limited geographical area. The cohort of considered subjects, however, evaluated with *a priori* power analysis, is characterized by a small sample size, suggesting that the obtained results should be addressed as preliminary observations; indeed, the significance obtained with the present data appears clear, although it may deserve a confirmation with larger sample size. Another limitation is the lack of some subject characteristics such as the duration of disease in patients with diabetes. As regards the correlations between metabolic and glyco-oxidation parameters, in particular those linked to HSA glycation and thiolation, a larger sample size would be needed to better define the type of relationships.

In conclusion, in patients with CHD, HSA glycation and S-thiolation have a different prevalent burden, with GLY-HSA prevailing in patients with T2DM and THIO-HSA in patients without T2DM. In particular, in T2DM patients, deleterious vascular effects could be originated from a complementary mechanism of action that includes higher levels of oxidative stress biomarkers, such as AGE, and the loss of albumin antioxidant capacity, suggesting how important is to take into consideration also albumin quality for the maintenance of homeostasis balance between glyco-oxidation and antioxidant defense system.

## Figures and Tables

**Table 1:** Glycation and oxidation parameters evaluated in the four groups of subjects.

Parameter	A: Diabetics with CHD (*n* = 33)	B: Diabetics without CHD (*n* = 31)	C: Non-diabetics with CHD (*n* = 30)	D: Controls (*n* = 27)
AGE (µg/mL)	7.8 ± 4.8 ^a^	6.7 ± 5.8 ^a^	2.6 ± 2.8 ^b^	2.3 ± 0.8 ^b^
Pentosidine (nmol/mL)	0.84 ± 0.24 ^a^	0.78 ± 0.23 ^a^	0.62 ± 0.22 ^b^	0.47 ± 0.08 ^c^
sRAGE (ng/mL)	0.95 ± 0.50 ^a^	0.62 ± 0.22 ^b^	0.79 ± 0.42 ^ab^	0.31 ± 0.07 ^c^
RAGE (pg/mL)	217 ± 171 ^a^	140 ± 61 ^b^	54 ± 33 ^c^	51 ± 13 ^c^
GLY-HSA (%)	13.2 ± 1.6 ^a^	13.1 ± 1.7 ^a^	12.1 ± 0.7 ^b^	12.02 ± 0.9 ^b^
THIO-HSA (%)	21.8 ± 2.9 ^a^	22.4 ± 2.9 ^a^	25.3 ± 3.7 ^b^	20.98 ± 3.4 ^a^

AGE, advanced glycation end products; GLY-HSA, Glycated albumin; RAGE, receptor for advanced glycation end-products; sRAGE, soluble RAGE; THIO-HSA, thiolated albumin; CHD, coronary heart disease. Data are expressed as mean ± SD. For each variable, means of groups not sharing any superscript letter are significantly different by Tukey’s HSD test at the 5% level of significance. Figure 2 indicates graphically the corresponding paired comparisons.

## Data Availability

Data collected in the study will be made available using the data repository Zenodo (https://zenodo.org/) with restricted access upon request to direzione.scientifica@ccfm.it.

## Supplementary Materials

The following supporting information can be downloaded at: https://www.mdpi.com/article/10.3390/antiox11081501/s1, Table S1. Clinical and metabolic data evaluated in the four groups of subjects. Table S2. Significant correlations between the metabolic and glyco-oxidation parameters evaluated considering all the patients (groups A, B, and C; *n* = 94).

## Abbreviations

AGE, advanced glycation end products; ANOVA, one-way analysis of variance; BMI, body mass index; CHD, coronary heart disease; CVD, cardiovascular diseases; DCF, 2,7 dichlorofluorescein; DCFH2-DA, 2′,7′-dichlorodihydrofluorescein diacetate; ELISA, enzyme-linked immunosorbent assay; FA, formic acid; FPG, fasting plasma glucose; GLY-HSA, glycated albumin; HbA1c, glycated hemoglobin; HDL, high-density lipoprotein; HF, heart failure; HSD, honest significance test; IFCC, International Federation of Clinical Chemistry; LC-MS, liquid chromatography-mass spectrometry; MS, mass spectrometry; LDL, low-density lipoprotein; NAC, N-acetyl-cysteine; RAGE, receptor for advanced glycation end-products; SD, standard deviation; sRAGE, soluble RAGE; T2DM, type 2 diabetes mellitus; THIO-HSA, thiolated albumin; TNFα, tumor necrosis factor-alpha.
