# Supplementary material for: The Burden of Impaired Serum Albumin Antioxidant Properties and Glyco-Oxidation in Coronary Heart Disease Patients with and without Type 2 Diabetes Mellitus
Source: Antioxidants (Basel). 2022 Jul 30;11(8):1501. doi: 10.3390/antiox11081501 (PMC9404962; doi:10.3390/antiox11081501)
Supplement: Supplementary file 1 [file antioxidants-11-01501-s001.zip › antioxidants-1809303-supplementary.pdf]

Supplementary Table S1. Clinical and metabolic data evaluated in the four groups of subjects.

Parameter	A - Diabetics with CHD (*n*=33)	B – Diabetics without CHD (*n*=31)	C - Non-diabetics with CHD (*n*=30)	D – Controls (*n*=27)
Age (y)	65.5±8.3[a]	60.0±5.9[a]	65.6±6.0[a]	47.3±13.4[b]
BMI (kg/m^2)	29.8±3.9[a]	29.2±3.4[ab]	26.8±5.1[bc]	25.5±3.3[c]
FPG (mg/dl)	142±41[a]	144±36[a]	91±11[b]	89±14[b]
HbA1c (%)	7.0±0.8[a]	6.9 ±0.8[a]	5.8±0.2[b]	5.5±0.4[b]
Total cholesterol (mg/dl)	151±35[b]	191±35[a]	155±40[b]	194±19[a]
LDL cholesterol (mg/dl)	82±30[b]	112±39[a]	86±31[b]	116±18[a]
HDL cholesterol (mg/dl)	42±9[b]	49±10[ab]	53±16[a]	56±13[a]
Triglycerides (mg/dl)	161±80[a]	138±72[a]	91±40[b]	93±28[b]

BMI, body mass index; CHD, coronary heart disease; FPG, fasting plasma glucose; GLY-HSA, glycated albumin; HbA1c, glycated hemoglobin; HDL, high density lipoprotein; LDL, low density lipoprotein. Data are expressed as mean ±SD. For each variable, means of groups not sharing any superscript letter are significantly different by the Tukey's HSD test at the 5% level of significance.

Supplementary Table S2. Significant correlations between the metabolic and glyco-oxidation parameters evaluated considering all the patients (groups A, B and C; *n*=94).

Parameter correlation	Pearson's *r*	*p*
AGE vs FPG	0.2955	0.0042
AGE vs HbA1c	0.2781	0.0073
AGE vs Pentosidine	0.2427	0.0198
AGE vs RAGE	0.3795	0.0002
RAGE vs sRAGE	0.5928	<0.0001
GLY-HSA vs FPG	0.3738	0.0002
GLY-HSA vs HbA1c	0.4903	<0.0001
GLY-HSA vs THIO-HSA	-0.3505	0.0005
THIO-HSA vs FPG	-0.3685	0.0003
THIO-HSA vs HbA1c	-0.3753	0.0002
THIO-HSA vs AGE†	0.3805	0.0289

AGE, advanced glycation end products; FPG, fasting plasma glucose; GLY-HSA, glycated albumin; HbA1c, glycated hemoglobin; RAGE, receptor for advanced glycation end-products; sRAGE, soluble RAGE; THIO-HSA, thiolated albumin. †The correlation THIO-HSA vs AGE is referred to Group A (Diabetics with CHD) only; no significant correlation was found for groups B and C.
